# Hemophagocytic Syndrome as an Initial Presentation of Systemic Lupus Erythematosus: A Case Report

**DOI:** 10.7759/cureus.5261

**Published:** 2019-07-29

**Authors:** Mohammed AlSheef, Abdul Rehman Z Zaidi, AbdulAziz A AlAtmi, Lina H AlSharif, Arshad Mian

**Affiliations:** 1 Medicine, King Fahad Medical City, Riyadh, SAU; 2 Internal Medicine, King Fahad Medical City, Riyadh, SAU; 3 Emergency Medicine, King Fahad Medical City, Riyadh, SAU

**Keywords:** hemophagocytic syndrome, systemic lupus erythematosus, middle east, sle, saudi arabia, hps

## Abstract

Hemophagocytic syndrome (HPS) is an uncommon potentially life-threatening hematological disorder characterized by fever, pancytopenia, lymphadenopathy, and activation of macrophages, which can be associated with various diseases. HPS brings significant diagnostic and therapeutic challenges, especially if it is the presenting manifestation of an autoimmune disorder, which is uncommon. We present a case of hemophagocytic syndrome as an initial presentation of systemic lupus erythematosus (SLE). We also highlight this rare initial presentation of SLE where initial antinuclear antibody and extractable nuclear antigen tests were negative. To the best of our knowledge, this is the first case of isolated HPS evolving into SLE in the Middle East.

## Introduction

Hemophagocytic syndrome (HPS) is an uncommon hematological disorder characterized by hyper-inflammatory response caused by abnormal activation of the immune system leading to an increase of cytokines in the blood [1]. HPS is a potentially life-threatening condition, which is usually manifested by fever, pancytopenia, lymphadenopathy, hepatosplenomegaly, elevated ferritin, and high triglycerides [2]. HPS has two types, primary (hereditary) and secondary (reactive). The secondary type is often associated with underlying disorders such as infections, malignancies, and autoimmune disorders, including systemic lupus erythematosus (SLE) [3]. When SLE is the fundamental cause of reactive hemophagocytosis, it is described as acute lupus hemophagocytic syndrome (ALHS) [4].

We report a rare presentation of SLE, which initially presented as HPS.

## Case presentation

A 24-year-old Saudi woman, not known to have any medical illness, presented to the emergency room with a history of high-grade fever for two weeks. She denied any history of joint pain, skin rash, oral ulcers, night sweats, headache, seizure, and weight loss. There was no family history of connective tissue diseases or malignancies.

**Figure 1:**
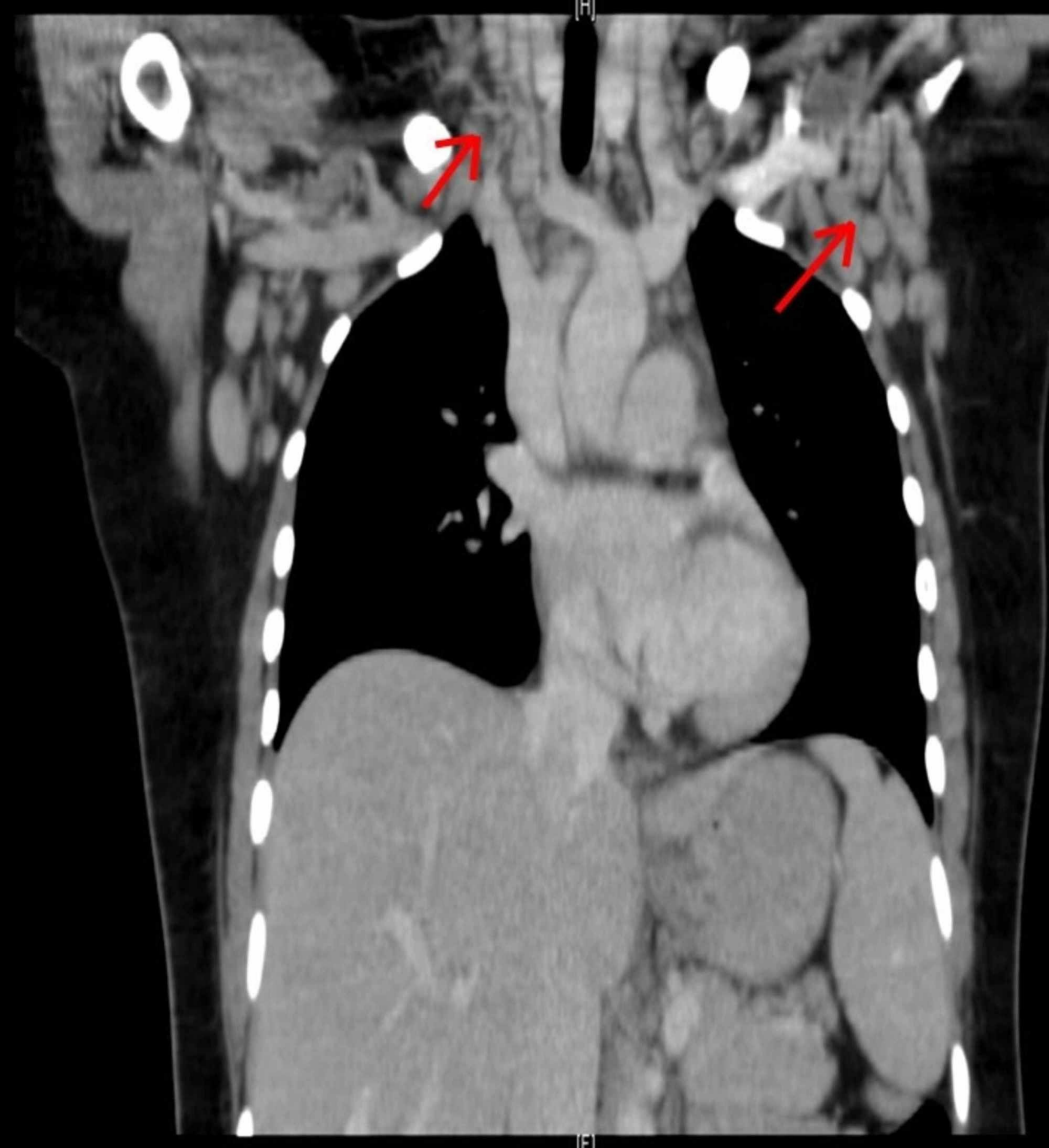
Coronal CT scan of the chest showed pathologically enlarged cervical, supraclavicular, axillary and mediastinal lymph nodes.

**Figure 2:**
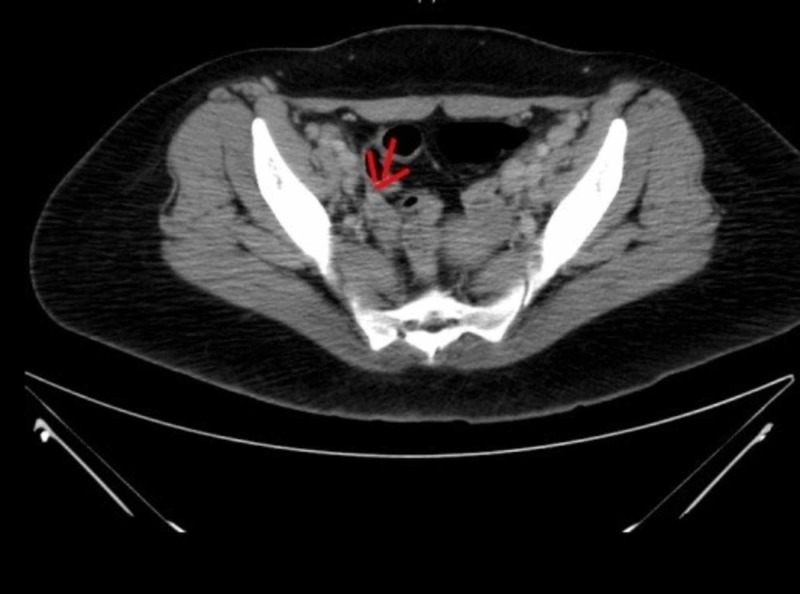
Axial CT scan of the pelvis showed pathologically enlarged inguinal lymph nodes.

**Figure 3:**
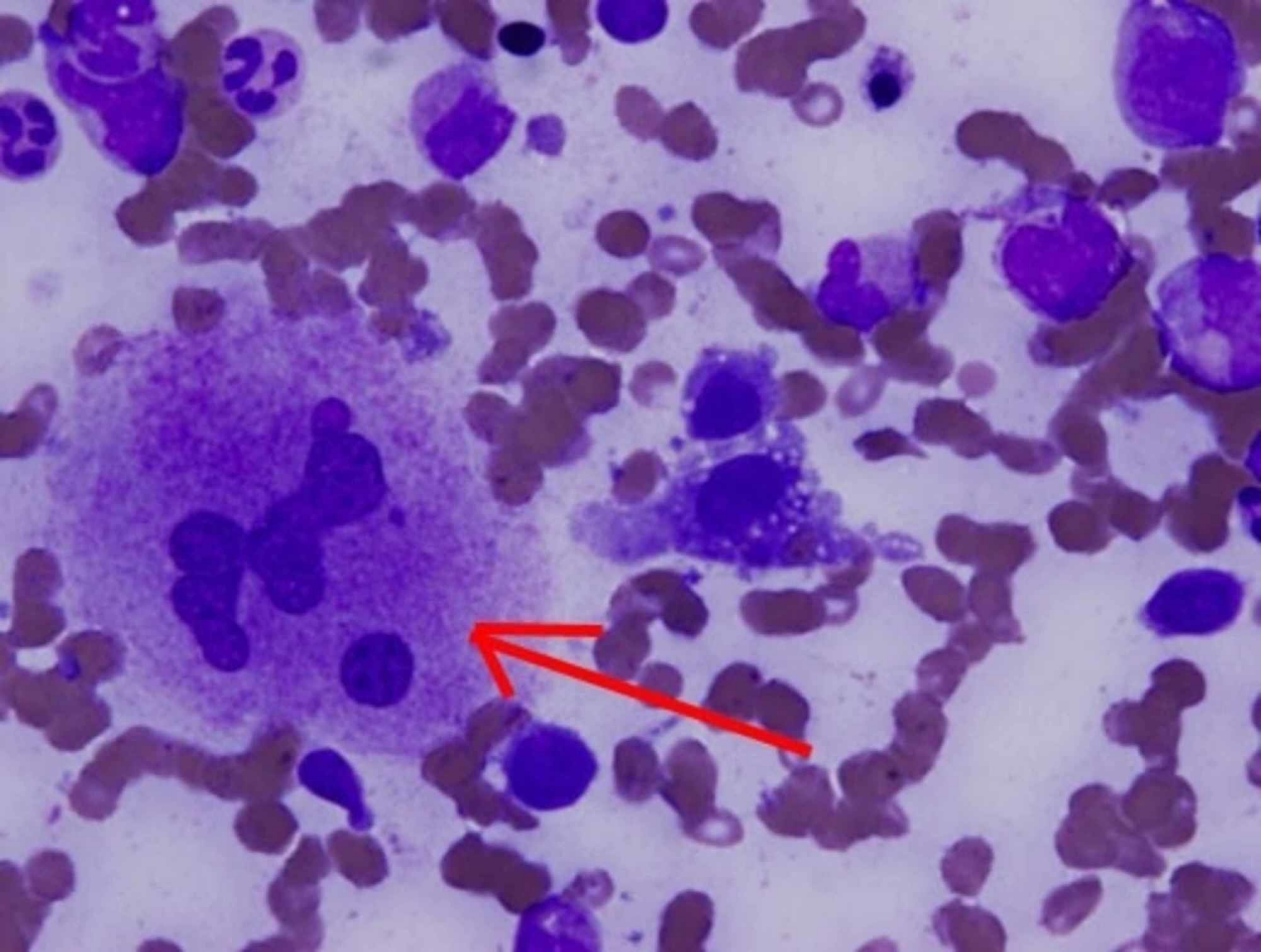
Bone marrow biopsy showed proliferation of macrophages with significant hemophagocytosis.

On examination, a palpable left posterior cervical lymph node (1 x 2 cm) was found. There was no organomegaly and no signs of active synovitis or skin rash. Laboratory examination revealed pancytopenia, coagulopathy and elevated liver function tests. Ferritin level was very high (5199 ug/L), antinuclear antibody test (ANA), and extractable nuclear antigen test (ENA) were initially negative. Peripheral blood film showed microcytic hypochromic anemia with 4% atypical lymphocytes. Erythrocyte sedimentation rate (ESR) was elevated (79 mm/h). C-reactive protein (CRP) was normal (3.9 mg/L). The culture was negative for fungi and bacteria. Serology was negative for cytomegalovirus (CMV), Epstein-Barr virus (EBV), hepatitis A virus (HAV), hepatitis B virus (HBV), hepatitis C virus (HCV) and human immunodeficiency virus (HIV). Computerized tomography (CT) scan showed multiple enlarged lymph nodes in both sides of the neck, supraclavicular, axillary, mediastinal, retroperitoneal, and pelvic area (Figures 1, 2). Bone marrow biopsy showed proliferation of macrophages with significant hemophagocytosis (Figure 3). Flow cytometry was negative for myeloproliferative and lymphoproliferative disorders.

After three weeks of extensive workup, no secondary cause for HPS could be identified (Table 1). Lymph node biopsy was refused by the patient. She was treated with general supportive measures including broad-spectrum antibiotics, blood products, and granulocyte colony-stimulating factor (GCSF) with significant clinical improvement. Five months later, on follow-up, she developed arthralgias, alopecia, and a malar rash. Repeated ANA/ENA screen was strongly positive. A diagnosis of SLE was made as per the American College of Rheumatology (ACR) criteria. Subsequently, she was started on prednisone and hydroxychloroquine and improved dramatically.

**Table 1:** A comparison between blood tests during patient’s initial presentation and follow-up visits. WBCs: White Blood Cells; ANC: Absolute Neutrophil Count; Hb: Hemoglobin; APTT: Activated Partial Thromboplastin Time; ANA: Antinuclear Antibody; Anti-ds DNA: Anti-double stranded DNA; AST: Aspartate Aminotransferase; ALT: Alanine Aminotransferase; ALP: Alkaline Phosphatase; LDH: Lactate Dehydrogenase.

Test	Value at Initial Presentation	Value at Follow-up Visits	Reference Range
WBCs	0.92 x 10^9^/L	3.75 x 10^9^/L	4-11 x 10^9^/L
ANC	0.19 x 10^9^/L	1.8 x 10^9^/L	2.6-7.5 x 10^9^/L
Hb	10.6 g/dl	13.1 g/dl	12-16 g/dl
Platelets	95 x 10^9^/L	164 x 10^9^/L	140-450 x 10^9^/L
Ferritin	5199 mg/L	148 mg/L	13-150 mg/L
APTT	70.2 sec.	36 sec.	26-40 sec.
ANA	Negative (twice)	Positive	
Anti-ds DNA	Negative	Positive (240)	
AST	934 U/L	35 U/L	15-37 U/L
ALT	194 U/L	34 U/L	30-66 U/L
ALP	642 U/L	71 U/L	50-136 U/L
Gamma GT	1669 U/L	20 U/L	7-32 U/L
LDH	4207 U/L	220 U/L	81-234 U/L
Triglycerides	2.79 mmol/L	1.59 mmol/L	0-1.7 mmol/L

## Discussion

In our case, all the clinical findings and laboratory results indicate the diagnosis of HPS initially without any provocative disease. Five months later, on follow-up, she developed arthralgias, alopecia, and a malar rash. Repeated ANA/ENA screen was strongly positive. According to the previous presentation, HPS preceded the evolution of full-blown SLE. In contrast, in 2011, in France, Elqatni et al. reported a case of abdominal pain that was associated with acute pancreatitis related to SLE, which was identified seven months before HPS [5]. However, in Japan, in the year 2012, Miura et al. reported a case of lupus nephritis complicated with nephrotic syndrome after extensive treatment and the disease had been controlled. In addition, their patient suffered from EBV that was associated with HPS and treated with chemotherapy and achieved complete remission [6]. Another case was reported in 2002 by Moriguchi et al. in Japan, who presented in 1998 with a diagnosis of HPS and SLE with increasing interleukin (IL)-6 and IL-1 beta; this case indicated that high cytokines could be present with HPS [7]. A similar presentation was found in a case reported in 2008 by Taki et al. in Japan, who presented as a case of SLE that presented with HPS as initial presentation [3]. Also, in 2007, Yeap et al. in Taiwan reported a case of a female that presented initially with HPS as the initial presentation of SLE [8]. This illustrates that HPS can precede, coincide, or follow the evolution of full-blown SLE, which is exceedingly rare but a potentially fatal disorder. In the right clinical setting, patients with HPS without a clear secondary cause should be followed for the development of symptoms and signs of secondary HPS such as SLE.

## Conclusions

In conclusion, to the best of our knowledge, this is the first reported case in the Middle East of an adult presenting as HPS that later evolved to SLE. Furthermore, we should consider following patients with isolated HPS and anticipate for signs and symptoms of secondary HPS, such as SLE. This also calls attention to the importance of vigilance and the need for robust follow-up of patients presenting with HPS, even when the patient does not primarily have a clinical picture of SLE.
